# Pharmacotherapies and Aortic Heme Oxygenase-1 Expression in Patients with Abdominal Aortic Aneurysm

**DOI:** 10.3390/antiox11091753

**Published:** 2022-09-04

**Authors:** Anja Hofmann, Bianca Hamann, Anna Klimova, Margarete Müglich, Steffen Wolk, Albert Busch, Frieda Frank, Pamela Sabarstinski, Marvin Kapalla, Josef Albin Nees, Coy Brunssen, David M. Poitz, Henning Morawietz, Christian Reeps

**Affiliations:** 1 Division of Vascular and Endovascular Surgery, Department of Visceral, Thoracic and Vascular Surgery, University Hospital and Faculty of Medicine, Technische Universität Dresden, Fetscherstr. 74, D-01307 Dresden, Germany; 2 National Center for Tumor Diseases, Partner Site Dresden, Institute for Medical Informatics and Biometry, Medical Faculty Carl Gustav Carus, Technische Universität Dresden, D-01307 Dresden, Germany; 3 Clinic for Internal Medicine, Asklepios-ASB Klinik Radeberg, D-01454 Radeberg, Germany; 4 Division of Vascular Endothelium and Microcirculation, Department of Medicine III, University Hospital Carl Gustav Carus Dresden, Technische Universität Dresden, D-01307 Dresden, Germany; 5 Institute for Clinical Chemistry and Laboratory Medicine, University Hospital and Faculty of Medicine, Technische Universität Dresden, D-01307 Dresden, Germany

**Keywords:** vascular biology, cardiovascular risk factors, pharmacotherapy, abdominal aortic aneurysm, heme oxygenase-1

## Abstract

Background: Treatment of cardiovascular risk factors slows the progression of small abdominal aortic aneurysms (AAA). Heme oxygenase-1 (HO-1) is a stress- and hemin-induced enzyme providing cytoprotection against oxidative stress when overexpressed. However, nothing is known about the effects of cardiometabolic standard therapies on HO-1 expression in aortic walls in patients with end-stage AAA. Methods: The effects of statins, angiotensin-converting enzyme (ACE) inhibitors, angiotensin II receptor blockers (ARBs), calcium channel blockers (CCBs), beta-blockers, diuretics, acetylsalicylic acid (ASA), and therapeutic anticoagulation on HO-1 mRNA and protein expressions were analyzed in AAA patients using multivariate logistic regression analysis and comparison of monotherapy. Results: Analysis of monotherapy revealed that HO-1 mRNA and protein expressions were higher in patients on diuretics and lower in patients on statin therapy. Tests on combinations of antihypertensive medications demonstrated that ACE inhibitors and diuretics, ARBs and diuretics, and beta-blockers and diuretics were associated with increase in HO-1 mRNA expression. ASA and therapeutic anticoagulation were not linked to HO-1 expression. Conclusion: Diuretics showed the strongest association with HO-1 expression, persisting even in combination with other antihypertensive medications. Hence, changes in aortic HO-1 expression in response to different medical therapies and their effects on vessel wall degeneration should be analyzed in future studies.

## 1. Introduction

Abdominal aortic aneurysm (AAA) is a vascular pathology characterized by pathological dilation. AAA initiation and progression involves accumulation of mononuclear inflammatory cells and increased local cytokine and chemokine production. Furthermore, matrix degradation, oxidative and hemodynamic stress, and loss of medial vascular smooth muscle cells (VSMC) are found [1]. The majority of AAAs are covered by an intraluminal thrombus (ILT). ILT is a highly active compartment, and its presence is associated with thinning of the vessel wall and possibly increased risk of rupture [2]. Surgical repair of AAA is indicated at an aortic diameter of 55 mm to prevent rupture [3]. 

Pharmacological treatment of cardiovascular and metabolic risk factors is known to reduce the progression of small-diameter aneurysms, underlining the possibilities of medical AAA stabilization [4]. Pharmacological management of patients with diagnosed AAA includes prescription of lipid-lowering statins, angiotensin II receptor blockers (ARBs), and angiotensin-converting enzyme (ACE) inhibitors as antihypertensive therapy. ACE inhibitors, ARBs, beta-adrenergic receptor blockers (beta-blocker), calcium channel blockers (CCBs), and diuretics are used as antihypertensive therapy [4]. It has been demonstrated that ACE inhibitors can reduce inflammation in AAA [3]. Statins have been shown to reduce cardiovascular mortality in AAA and to slow AAA progression in preclinical animal models [5]. Although most of these drugs exert beneficial effects on the cardiovascular risk profile [4], a clear role in the stabilization of AAA has not been demonstrated.

Heme oxygenase-1 (HO-1) is a stress-induced enzyme that catalyzes the degradation of pro-oxidative heme proteins, thereby interfering with inflammation, oxidative stress, antioxidant functions, apoptosis, proliferation, thrombosis, and angiogenesis [6,7,8]. Overexpression of HO-1 gene provides cytoprotection against oxidative stress [9], and the beneficial effects of HO-1 induction have been described in various cardiovascular diseases [10]. The genetic deletion of HO-1 exacerbated the development of AAA in mouse models [11,12]. We have recently shown that HO-1 expression was increased in human late-stage AAA and that the increase was inversely linked to oxidative stress [13]. Mice lacking HO-1 had increased thrombus formation in response to vessel injury [14]. However, current knowledge on HO-1 expression and activity in response to pharmacotherapies is scarce and mainly derived from the analysis of single monotherapy in one cell type or from preclinical animal models. Targeting oxidative, degenerative, and inflammatory processes in the aortic wall and limiting thrombus formation by an induction of HO-1 may present an interesting target in AAA.

We hypothesized that patients on lipid-lowering and antihypertensive medications as well as therapeutic anticoagulation would have increased aortic HO-1 mRNA and protein expressions in late-stage AAA that is connected to a lowering of the AAA diameter and in the thickness of the ILT. Herein, we show that diuretics alone and in combination with ARBs, ACE, and beta-blockers increased HO-1 mRNA and protein expressions, while statin intake lowered HO-1 protein expression. Therapeutic anticoagulation and ASA had no clear effect, and HO-1 expression was not linked to the AAA diameter and thickness of the ILT.

## 2. Materials and Methods

### 2.1. Primary Outcome Variable 

Aortic HO-1 mRNA and protein expressions were set as the primary outcome variables, and their expressions were analyzed in response to prescription of different medical therapies. The following sections describe their quantification.

### 2.2. Abdominal Aortic Wall Acquisition and Patient Data

A total of 36 patients with diagnosed AAA were included in this consecutive study. Inclusion criteria were aortic diameter >50 mm, fast-growing AAA with >10 mm progress per year, or symptomatic AAA. Two patients had an aortic diameter >40 mm but below 50 mm and were treated for rapidly growing iliac artery aneurysm. Aortic tissues were collected intraoperatively during open surgical repairs from the anterior wall of the AAA sac between March 2017 and March 2022. Specimen were collected 10 min after removal, rinsed in ice-cold 1xDPBS, and cleaned from adjacent thrombus and blood clots. Tissue for RNA analysis was immediately frozen in liquid nitrogen. HO-1 mRNA expression was measured in *n* = 31 specimen, and protein expression was measured in *n* = 29 specimen. Due to the low quality of RNA or protein, qPCR and Western blot were not performed in the same samples, and *n* = 11 AAA did not match in mRNA and gene expression. This analysis included two patients where two specimen were obtained from an anatomically proximal region of the AAA. However, these specimens were handled as individual samples to avoid calculating the mean. The mean aortic diameter was assessed by computed tomography (CT) angiography prior to the surgical procedure by measuring the outer–outer diameter. The thickness of the ILT was determined in the arterial phase in CT scans after multiplanar reconstruction. The aorta was scanned in an axial position in 1 mm sections, and the thickness of the ILT was measured at the largest distance from the inner surface of the lumen to the outer aortic wall. Information on sex was self-reported, and the distribution is given in the description of baseline demographics. Blood lipids, C-reactive protein (CRP), cardiovascular risk factors, comorbidities, and medical therapies were evaluated retrospectively. Smoking was defined as presently smoking or any kind of smoking history.

### 2.3. Pharmacological Therapies

The pharmacological therapies included statins (atorvastatin, simvastatin, and rosuvastatin), ARBs (losartan, valsartan, and candesartan), ACE inhibitors (ramipril, lisinopril, enalapril, and perindopril), anticoagulants (enoxaparin sodium: 40 mg; rivaroxaban: 15 mg; apixaban: 2.5, 5 mg; and phenprocoumon: 3 mg), acetylsalicylic acid (aspirin (ASA): 100 mg), CCB (amlodipine: 5, 10 mg; nifedipine: 20 mg; and lercanidipine: 10, 20 mg), beta-blockers (bisprolol, metoprolol, nebivolol, carvedilol, and propranolol), and diuretics (hydrochlorothiazide (HCT): 12.5, 25 mg; furosemide: 40, 250 mg; torasemide: 5, 10, 20 mg; and indapamide: 1.25 mg). A detailed description of pharmacological treatments with statins, ARBs, ACE inhibitors and beta--blockers with corresponding doses is given in Table S1.

### 2.4. RNA Isolation, cDNA Synthesis, and Quantitative Real-Time PCR (qPCR)

Aortic tissue (30–50 mg) was homogenized in 1 mL TriFast (VWR, Darmstadt, Germany) using a Precellys 24 homogenizer (VWR, Darmstadt, Germany), and RNA was isolated according to the manufacturer’s instructions. Afterwards, an RNA clean and concentrator kit (R1016, Zymo Research, Freiburg im Breisgau, Germany) was used with an additional on-column DNase I digestion to remove the remaining DNA. Reverse transcription of mRNA into cDNA was performed with MultiScribe reverse transcriptase (Thermo Fisher Scientific, Darmstadt, Germany) using random hexamer primers according to the manufacturer’s instructions. Quantification of mRNA expression was performed by real-time PCR with GoTaq qPCR master mix (Promega, Walldorf, Germany) and the Step One Plus Real-Time PCR system (Thermo Fisher Scientific, Darmstadt, Germany) [13]. A geometric mean of three reference genes, namely, ribosomal protein L32 (*RPL32*), TATA-box binding protein (*TBP*), and beta-2-microglobulin (*B2M*), was used for cDNA content normalization [15]. To ensure comparability of data, an internal control was run in every reverse transcription, and mRNA expression was set to 1. Data are presented as ΔCT values in relation to the internal control. Primer sequences were as follows: NM_002133.2, *HO-1*-sense: AGTCTTCGCCCCTGTCTACT, *HO-1*-antisense CTTCACATAGCGCTGCATGG; NM_000994.4, *RPL32*-sense CCACCGTCCCTTCTCTCTTC; *RPL32*-antisense GCTTCACAAGGGGTCTGAGG; NM_003194.5, *TBP*-sense CGCCGGCTGTTTAACTTCG, *TBP*-antisense AGAGCATCTCCAGCACACTC, *B2M*-sense: GATGAGTATGCCTGCCGTGT; *B2M*-antisense: CATGATGCTGCTTACATGTCTCG.

### 2.5. Protein Isolation and Western Blot

Aortic tissues were grounded in liquid nitrogen using a mortar and lysed in 1xRIPA buffer (10 mg/100 µL) supplemented with 1:100 Halt protease and phosphatase inhibitor cocktail (Thermo Fisher Scientific, Darmstadt, Germany). Ultrasonication (15 s) was applied to remove remaining DNA, and protein concentration was determined by BCA protein assay reagent (Thermo Fisher Scientific, Darmstadt, Germany). Proteins (15–30 µg) were separated by 4–12% Bis-Tris protein gels (Thermo Fisher Scientific, Darmstadt, Germany) and transferred to nitrocellulose membranes [13]. Membranes were incubated with a primary antibody against HO-1 using a dilution of 1:500–1:1000 (#610713, BD Biosciences, Heidelberg, Germany). Protein expression was detected with Immobilon Western HRP substrate (Merck, Darmstadt, Germany). Two bands obtained at ~32 and ~25 kDa were quantified for expression analysis. The upper band has been shown to be the complete protein, while the lower is a truncated version [16]. The truncated version is described to be enzymatically inactive but is known to induce oxidant-responsive transcription factors [16]. The band intensity was quantified using Image J software. Protein expression was normalized to a 70 kDa band obtained after Ponceau S staining because other tested reference proteins showed a strong variation within the group (data not shown). To ensure comparability between different western blots, a standardized internal control was run on every gel and protein expression was normalized to this control (=1).

### 2.6. Statistical Analyses

Grubb’s outlier test was used to detect significant outliers, and normality was tested by the D’Agostino and Pearson test. HO-1 mRNA and protein expressions were log transformed and showed a continuous distribution. Multiple logistic regression analysis was used to test the effects of pharmacological therapies on the primary outcome variables. Different combinations of antihypertensive therapies were tested and chosen based on the recommendations of the ESC [17]. Combinations were as follows: ACE inhibition and diuretics, ARBs and diuretics; ACE inhibitors and CCB; ARBs and CCB; beta-blocker and diuretics; CCB and beta-blockers; and triple therapy of CCB, ACE inhibitors, and diuretics. In addition, AAA risk factors, namely, age, hypertension, smoking, coronary artery disease (CAD), and total cholesterol levels, were analyzed for their effects on the outcome variables. Pharmacological therapies and risk factors were given as categorical numbers (0 = no treatment, 1 = on treatment). Stepwise model selection using penalized multiple linear regression was used to assess the potential influence of medical therapies on HO-1 mRNA or protein expression. R studio software (Boston, MA, USA) was used for multiple linear regression and weighted linear regression analysis. Effects of a single pharmacotherapy were tested, and non-Gaussian distributed data were compared by Mann–Whitney *U* test, normally distributed by unpaired *t*-test. Depending on normality distribution, data are shown as scatter dot plots, where the horizontal line depicts the mean. If normal distribution was violated, data are presented as median. Graph Pad Prism 9.0 (GraphPad Software, Inc., La Jolla, CA, USA) software was used for statistical analysis, and *p* < 0.05 was considered as significant.

## 3. Results

### 3.1. Demographic Characteristics of Analyzed AAA Patients

The mean age and aortic diameter of these patients at the time of surgery was 65.3 ± 7.4 years and 62.8 ± 13.9 mm, respectively. Of these patients, 72% received statins, 50% beta-blockers, 47% ACE inhibitors, 42% CCB, 36% diuretics, and 33% ARBs. Aspirin (ASA) was administered to 56% of patients, and 22% received therapeutic anticoagulation. A detailed description of baseline demographics, risk factors, and pharmacological therapies is given in Table S2.

### 3.2. Effects of AAA Risk Factors on Aortic HO-1 mRNA and Protein Expressions

The AAA risk factors, namely, age, hypertension, smoking, CAD, and total cholesterol, were not linked to HO-1 mRNA and protein expressions. Interestingly, expressions of HO-1 mRNA and protein showed completely opposite trends in patients with smoking and CAD. While HO-1 mRNA expression was lowered in AAA patients with smoking or CAD, HO-1 protein was increased; both changes did not reach significance.

### 3.3. Hierarchy of Treatments Affecting HO-1 Expression

**Figure 1:**
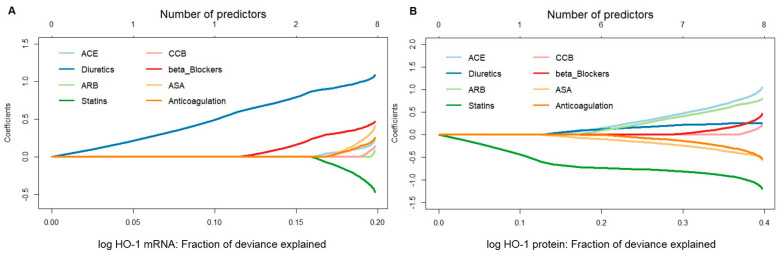
Stepwise model selection using penalized multiple linear regression for HO-1 mRNA and protein expressions in response to different pharmacological therapies in aortic tissues obtained from patients undergoing elective open surgical repair due to AAA. (**A**) mRNA expression was measured by qPCR, and data were normalized to an internal control (=1). (**B**) Protein expression was measured by Western blot, and data were normalized to an internal control (=1). (**A**,**B**) Data for HO-1 expression were log transformed, and one statistically significant outlier was omitted from the analysis. Data were analyzed by penalized multiple linear regression (elastic net approach), and HO-1 was set as the outcome variable. Predictors were added to a regression model one at a time to maximize the deviance (*x*-axis) given the current number of predictors. Regression coefficients, shown on the *y*-axis, changed as a new predictor was added to the model and reflected the influence of each predictor depending on other predictors present in the model. Abbreviations: ACE, angiotensin-converting enzyme; ARB, angiotensin II receptor blockers; ASA, acetylsalicylic acid; CCB, calcium channel blockers.

A penalized linear regression model fitted by the elastic-net method was used to assess the potential influence of medical therapies, namely, ACE, anticoagulation, ASA, ARB, beta-blockers, CCB, diuretics, and statins, on HO-1 mRNA or protein expression. Fit results indicated that, for HO-1 mRNA, the effect estimate for diuretics was persistently large, both when used as a single predictor and in combination. Patients taking diuretics were found to have much higher HO-1 mRNA expression values than those who did not. Beta-blockers and statins were found to be the second and third most important variables associated with HO-1 mRNA. On the other hand, for HO-1 protein values, statins played a major role, both as a single predictor and in combination, leading to lower expression values in patients on this therapy (Figure 1A,B).

### 3.4. Regulation of HO-1 mRNA and Protein Expressions in Response to Cardiometabolic Standard Therapies

**Figure 2:**
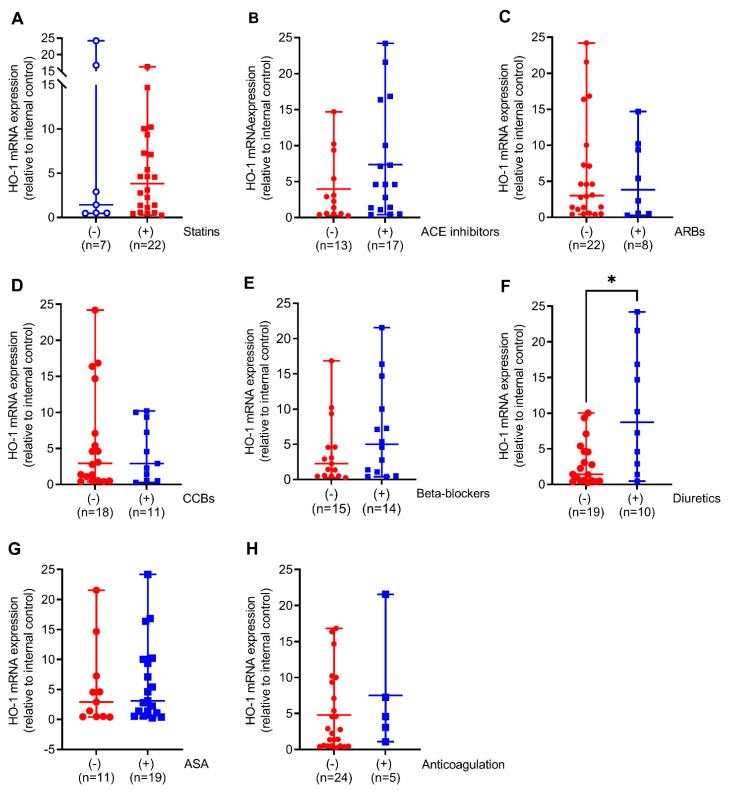
Comparison of HO-1 mRNA expression in response to different pharmacological therapies in aortic tissues obtained from patients undergoing elective open surgical repair due to AAA. (**A**–**H**) mRNA expression was measured by real-time PCR, and data were normalized to an internal control that was set to 1. Patients were divided according to the presence (+, blue) or absence (−, red) of the indicated therapy. Statistics: Statistically significant outliers were detected by Grubb’s outlier test. Data are presented as scatter dot plots, where the horizontal line depicts the mean or median with range depending on normality testing. (**B**,**C**) Unpaired *t*-test. (**A**,**D**–**H**) Mann–Whitney *U* test. * *p* < 0.05 (+) vs. (−) treatment. (**A**,**D**–**F**,**H**) One significant outlier was detected in each group. (**B**,**G**) One significant outlier was detected in the (−) group. (**C**) One significant outlier was detected in the (+) group. Abbreviations: ACE, angiotensin-converting enzyme; ARB, angiotensin II receptor blockers; ASA, acetylsalicylic acid; CCB, calcium channel blocker.

**Figure 3:**
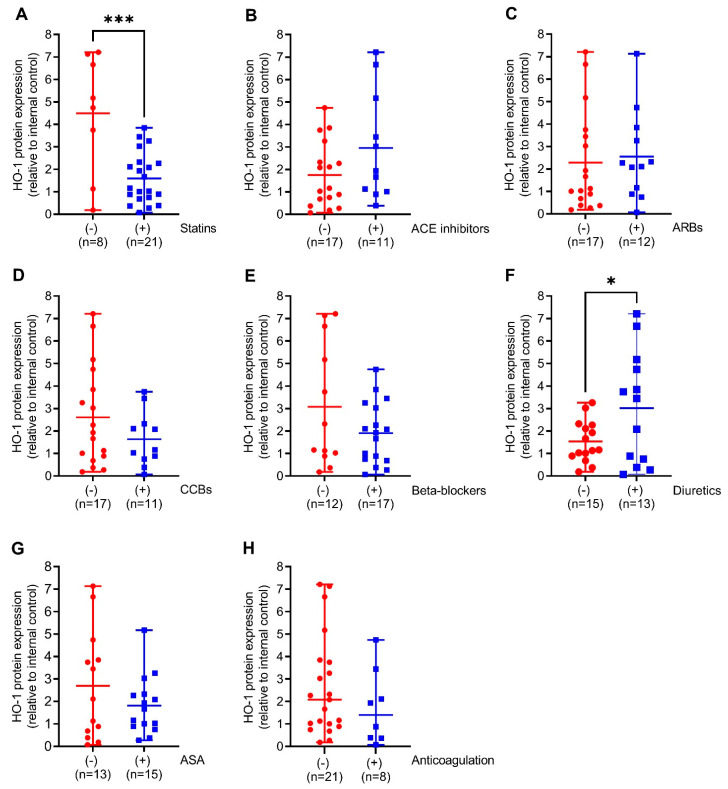
Comparison of HO-1 protein expression in response to different pharmacological therapies in aortic tissues obtained from patients undergoing open surgical repair due to AAA. (**A**–**H**) Protein expression was measured by Western blot, and results were divided into patients receiving the indicated therapy (+, blue) or not (−, red). (**A**) 70 kDa band obtained after Ponceau S staining served as the loading control, and data were normalized to an internal control (=1). Statistics: Significant outliers were detected by Grubb’s outlier test and were removed from all further analysis. (**B**) One significant outlier was removed from the (−) group. (**D**,**F**) One significant outlier was removed from the (+) group. Data are presented as scatter dot plots, where the horizontal line depicts the mean or median with range depending on normality testing. (**A**–**F**), Unpaired *t*-test. (**G**,**H**) Mann–Whitney *U* test. * *p* < 0.05 (−) vs. (+) diuretics. *** *p* < 0.001 (−) vs. (+) statins. Abbreviations: ACE, angiotensin-converting enzyme; ARB, angiotensin II receptor blockers; ASA, acetylsalicylic acid; CCB, calcium channel blocker.

We next compared HO-1 mRNA expression in the aortic walls of AAA patients with (+) or without (−) prescription of the indicated medical therapies. Patients on diuretics had 6-fold (*p* < 0.05) increased HO-1 mRNA expression. This was partly found in patients on ACE inhibitors and beta-blockers but not on other pharmacological therapies (Figure 2A–F). Similar to HO-1 mRNA expression, HO-1 protein expression was 2-fold higher (*p* < 0.05) in patients receiving diuretics but, surprisingly, 2.7-fold decreased (*p* < 0.001) under prescription of statins. Patients receiving ACE inhibitors, beta-blockers, and CCBs had increased HO-1 protein expression without reaching significance (Figure 3A–F).

### 3.5. Regulation of Aortic HO-1 Expression towards Combinations of Antihypertensive Treatments

Different combinations of antihypertensive therapies and their effects on aortic HO-1 mRNA and protein expressions were tested to assess additive effects. Combinations were as follows: ACE inhibition and diuretics; ARBs and diuretics; ACE inhibitors and CCB; ARBs and CCB; beta-blocker and diuretics; CCB and beta-blockers; and triple therapy of CCB, ACE inhibitors, and diuretics. Patients with prescription of ACE inhibitors and diuretics, ARBs and diuretics, and beta-blockers and diuretics had an increased HO-1 mRNA expression. This was also found in patients with ACE, CCB, and diuretics. Combinations without prescription of diuretics revealed no effect on HO-1 mRNA expression (Table 1). HO-1 protein expression showed a similar trend in response to prescription of the indicated treatments, without reaching significance (Table 2).

### 3.6. Linkage of HO-1 Expression with AAA Diameter and Thickness of the Intraluminal Thrombus

HO-1 expression was connected to the AAA diameter and the ILT thickness, both important parameters of individual patient rupture risk. Because the ILT is known to affect the AAA diameter, these two parameters were tested simultaneously [2]. Due to the experimental design of the study, we assumed that levels of HO-1 mRNA and protein expressions were a consequence of the aortic diameter and the thickness of the ILT. However, both did not affect aortic HO-1 expression, suggesting that expressions of HO-1 within the AAA walls may not be linked directly to increase in diameter (Table 3).

## 4. Discussion

In the present study, aortic HO-1 mRNA and protein expressions in response to prescription of lipid-lowering and antihypertensive therapies as well as therapeutic anticoagulation were analyzed in aortic tissues of patients with late-stage AAA. These therapies were tested for their potential to induce HO-1 expression because an induction of HO-1 is considered to be protective in many cardiovascular diseases, including AAA [10,11,12,13].

In this study, we showed that HO-1 mRNA and protein expressions was higher in AAA patients on prescription of diuretics. In contrast, those receiving statins revealed lower HO-1 expression. Testing combinations of antihypertensive medications demonstrated that patients on ACE inhibitors and diuretics, ARBs and diuretics, and beta-blockers and diuretics had increased HO-1 mRNA expression. This was also found for the triple combination of ACE, CCB, and diuretics. Interestingly, HO-1 protein expression showed similar trends without reaching significance. HO-1 expression is mainly regulated on the transcriptional level [18], and differences in the regulation of HO-1 mRNA and protein expressions were found within the present study, mainly when adjusting for the presence of smoking and CAD as comorbidities. It has been shown that hemin- and stress-mediated HO-1 induction are regulated in two distinct ways [19]. Herein, different stressors could have contributed to the overserved regulation. In addition, HO-1 gene polymorphisms [20], posttranslational mechanisms [21], or interference of medical therapies with HO-1-regulating signaling pathways may have contributed to the present findings.

In the present study, 38% of all patients received diuretics, which are recommended as the first-line option in antihypertensive treatments [17]. Diuretics encompass thiazides and loop diuretics, which reduce arterial blood pressure by decreasing renal sodium reabsorption [17,22]. Previous studies have demonstrated that indapamide, a thiazide-type diuretic, combines a mild diuretic activity with direct vasodilatory effects that are due to activation of vascular potassium channels [23]. An induction in HO-1 has been shown to mediate endothelium-dependent hyperpolarization, thus contributing to enhanced arterial vasodilation in spontaneously hypertensive rats [24]. Eventually, this could have contributed to the induction of HO-1 expression in the present study. 

Clinically recommended combinations of antihypertensive medications [17] were tested to obtain information on the additive effects on HO-1 expression. Of interest, increase in HO-1 expression in patients on diuretics was independent of ARB, ACE, or beta-blocker prescription, suggesting that increased HO-1 mRNA and protein expressions are independent of other antihypertensive medications. No additive effects were observed for CCB and RAAS inhibition and CCB with beta-blockers. The increase caused by diuretics could be due to a lowering in blood pressure or as a feedback regulation in response to increased urinary HO-1 excretion. The molecular mass of HO-1 is low, and higher urinary excretion [25] with subsequent release from HO-1-expressing cells in the body is likely. Furthermore, prolonged HO-1 activation was shown to be protective in renovascular hypertension by counteracting the RAAS [26].

ACE inhibitors and ARBs are the most widely used class of antihypertensive drugs targeting the renin–angiotensin–aldosterone system (RAAS) [17]. Herein, AAA patients with prescription of ACE inhibitors and ARBs showed a slight but not significant increase in HO-1 expression. Active metabolites of ramipril, an ACE inhibitor, increased HO-1 in endothelial cells [27]. In contrast, Ang II decreased HO-1 in vascular smooth muscle cells, while the ARB losartan prevented HO-1 reduction [28]. The increase in HO-1 in the present study could be due to general reduction in blood pressure or reduced Ang II-mediated oxidative stress by the inhibition of RAAS components [29,30]. The ARB telmisartan could reduce blood pressure but not aneurysm growth in AAA patients [31].

Interestingly, HO-1 protein was significantly reduced in patients with prescription of statins. Simvastatin was shown to induce HO-1 in vascular smooth muscle cells [32] as part of a post-transcriptional mechanism that increases mRNA stability [18]. However, HO-1 protein was decreased in stable atherosclerotic plaques obtained from patients treated with simvastatin [33], supporting the findings of the present study. The decrease in HO-1 expression herein could be due to changes in ubiquitination or proteasomal degradation [34] or by the direct effects on HO-1-expressing cells, e.g., macrophages.

The majority of AAAs are covered by an intraluminal thrombus (ILT) that contributes to aortic wall degeneration [2]. Deletion of HO-1 accelerates arterial thrombus formation in response to oxidative stress [35]. Herein, we showed no linkage of the ILT thickness and aortic diameter with HO-1 mRNA and protein expression. This could be due to the analysis of end-stage AAA while animal studies mostly use young and male mice. Nonetheless, HO-1 can be protective in AAA initiation and progression by interfering with oxidative stress, inflammation, or degenerative mechanisms [6,36], which all contribute to degeneration of the vessel wall and increase in aortic diameter. Furthermore, we assumed that HO-1 expression is a consequence of the AAA diameter and ILT, but the AAA diameter and ILT thickness could have been affected by HO-1 as well.

An induction of HO-1 is known to reduce thrombus formation in mice [37], and therefore the effects of blood thinning and antithrombotic medications on HO-1 expression were analyzed. Weighted linear regression revealed a lowering in HO-1 protein expression in patients with ASA and anticoagulation, suggesting that prescription of these medications might have affected HO-1 expression by reducing, for example, oxidative stress arising from the ILT. Further studies have to analyze whether prescription of other lipid-lowering or antihypertensive treatments have synergistic or opposing effects and whether the reduction in HO-1 is protective. Next to this, the onset of ILT formation might be earlier in disease progression than the patients’ first prescription of ASA or therapeutic anticoagulation.

## 5. Limitations of the Present Study

The present study is a retrospective and descriptive study that does not allow conclusions on detailed mechanisms and direct effects of pharmacological treatments at, for example, the single cell level. Analysis of AAA samples at the late stage of disease may have limited relevance for the pathogenic processes because pharmacological therapies are probably more protective in earlier stages of the disease. In addition, the number of analyzed aortic tissues was limited, and analyzing HO-1 protein expression does not allow conclusions on enzymatic HO-1 activity. A subgroup analysis of different substance classes would be desirable but requires a sufficient number of analyzed patients. It could be that different types of statins (e.g., atorvastatin and simvastatin) or different doses might have different effects due to their pharmacology [32]. Finally, the lack of data on mRNA and protein expressions in the same AAA sample could have contributed to different regulations of mRNA and protein expressions.

## 6. Conclusions

The present study demonstrated that prescription of diuretics alone or in combination with antihypertensive ARBs, ACE inhibitors, and beta-blockers was significantly associated with increase in aneurysmal HO-1 expression, which partly refutes our initial contradictory hypothesis. In contrast, lipid-lowering statin therapy was associated with reduction in HO-1 expression. Changes in aneurysmal HO-1 expression were not affected by the aortic diameter or thickness of the ILT. The present study could be useful in designing further studies that may assess the effects of single or combination therapies in preclinical AAA models in a cell-type-specific manner and by analyzing disease-specific mechanisms. In summary, the changes in aortic HO-1 mRNA and protein expressions linked to prescription of medical therapies should be considered under the light of mechanisms contributing to vessel wall degeneration in AAA. Furthermore, additive and opposing effects of pharmacological therapies have to be carefully analyzed.

## Figures and Tables

**Table 1:** Aortic HO-1 mRNA expression in response to prescription of different combinations of antihypertensive therapies in patients undergoing open surgical repair due to AAA. Data were analyzed by multiple linear regression, and HO-1 was set as the outcome variable. Data were log transformed, and one statistically significant outlier was omitted from the analysis. The estimate shows the proportional increase in HO-1 mRNA expression if the patient received the indicated therapy, whereas those that did not receive therapy serve as the reference level (=0). Abbreviations: ACE, angiotensin-converting enzyme; ARB, angiotensin II receptor blockers, CCB, calcium channel blockers, CI, confidence interval.

log HO-1 mRNA				
Treatment combination	Estimate	CI left	CI right	*p*-value
ARBs [0]	1.024	0.410	2.559	0.960
Diuretics [0]	3.144	1.315	7.512	0.014
ACE [0]	1.192	0.536	2.651	0.670
Diuretics [0]	3.060	1.290	7.259	0.015
ARBs [0]	0.861	0.312	2.373	0.773
CCBs [0]	0.919	0.357	2.367	0.862
CCBs [0]	0.923	0.369	2.306	0.865
ACE [0]	1.339	0.564	3.178	0.511
Beta-blockers [0]	1.486	0.663	3.330	0.342
Diuretics [0]	2.844	1.189	6.800	0.024
CCBs [0]	1.862	0.785	4.420	0.166
Beta-blockers [0]	1.034	0.414	2.584	0.943
ACE [0]	1.183	0.523	2.678	0.689
CCBs [0]	0.944	0.399	2.229	0.896
Diuretics [0]	3.056	1.274	7.333	0.017

**Table 2:** Aortic HO-1 protein expression in response to prescription of different combinations of antihypertensive therapies in patients undergoing open surgical repair due to AAA. Data were analyzed by multiple linear regression, and HO-1 was set as the outcome variable. Data were log transformed, and one statistically significant outlier was omitted from the analysis. The estimate shows the proportional increase in HO-1 protein expression if the patient received the indicated therapy, whereas those that did not receive therapy serve as the reference level (=0). Abbreviations: ACE, angiotensin-converting enzyme; ARB, angiotensin II receptor blockers, CCB, calcium channel blockers, CI, confidence interval.

log HO-1 Protein				
Treatment combination	Estimate	CI left	CI right	*p*-value
ARBs [0]	1.695	0.617	4.657	0.314
Diuretics [0]	2.068	0.768	5.573	0.161
ACE [0]	1.617	0.608	4.300	0.344
Diuretics [0]	1.778	0.659	4.797	0.265
CCBs [0]	1.314	0.469	3.682	0.607
ACE [0]	1.879	0.680	5.187	0.233
ARBs [0]	1.545	0.503	4.747	0.454
CCBs [0]	0.950	0.316	2.858	0.928
Beta-blockers [0]	0.948	0.352	2.549	0.916
Diuretics [0]	1.933	0.697	5.359	0.215
Beta-blockers [0]	1.090	0.404	2.945	0.866
CCBs [0]	1.127	0.405	3.140	0.820
ACE [0]	1.708	0.611	4.775	0.316
CCBs [0]	1.237	0.440	3.477	0.690
Diuretics [0]	1.738	0.631	4.788	0.294

**Table 3:** Aortic HO-1 mRNA and protein expressions in response to the aortic diameter and the thickness of the intraluminal thrombus (ILT). Data were analyzed by multiple linear regression, and HO-1 mRNA or protein expression was set as the outcome variable. Data for HO-1 were log transformed, and one statistically significant outlier each was omitted from the analysis for mRNA and protein. The estimate represents the proportional increase in HO-1 mRNA and protein expressions with the increase in aortic diameter and ILT thickness per one millimeter. Abbreviations: CI, confidence interval.

log HO-1 mRNA				
Parameter	Estimate	CI left	CI right	*p*-value
Aortic diameter, mm	1.015	0.976	1.054	0.467
Thickness ILT, mm	0.983	0.936	1.032	0.488
**log HO-1 protein**				
Parameter	Estimate	CI left	CI right	*p*-value
Aortic diameter, mm	0.981	0.943	1.020	0.333
Thickness ILT, mm	1.010	0.962	1.061	0.680

## Data Availability

Not applicable.

## Supplementary Materials

The following supporting information can be downloaded at: https://www.mdpi.com/article/10.3390/antiox11091753/s1, Table S1: Summary of included pharmacological treatments and corresponding doses; Table S2: Clinical characteristics and baseline demographics of included patients with diagnosed AAA; Table S3: Aortic HO-1 mRNA expression in response to cardiovascular risk factors in patients undergoing open surgical repair due to AAA; Table S4: Aortic HO-1 protein expression in response to cardiovascular risk factors in patients undergoing open surgical repair due to AAA.
