# Supplementary material for: Pharmacotherapies and Aortic Heme Oxygenase-1 Expression in Patients with Abdominal Aortic Aneurysm
Source: Antioxidants (Basel). 2022 Sep 4;11(9):1753. doi: 10.3390/antiox11091753 (PMC9495607; doi:10.3390/antiox11091753)
Supplement: Supplementary file 1 [file antioxidants-11-01753-s001.zip › antioxidants-1874117-supplementary.pdf]

Table S1. Summary of included pharmacological treatments and corresponding doses. All doses are given in milligrams. Abbreviations: ACE, angiotensin-converting enzyme; ARBs, angiotensin receptor blocker; CCBs, calcium channel blockers.

**ARBs**	Dose (mg)
Valsartan	5, 51.4, 40, 160, 320
Candesartan	4, 8, 16, 32
**ACE inhibitors**	Dose (mg)
Ramipril	2.5, 5, 7.5, 1
Lisinopril	10, 20
Enalapril	10
**Statins**	Dose (mg)
Atorvastatin	10, 20, 40, 80
Simvastatin	5, 10, 20, 30, 40, 80
Rosuvastatin	40
**Beta-blockers**	Dose (mg)
Bisprolol	2.5
Metoprolol	23.75, 47.5, 85 200
Nebivolol	5
Carvedilol	6.25, 25
Propranolol	40

Table S2. Clinical characteristics and baseline demographics of included patients with diagnosed AAA. Blood glucose, triglycerides, CRP, HDL, LDL, and total cholesterol were measured in the Institute for Clinical Chemistry and Laboratory Medicine at TU Dresden. Data were analyzed retrospectively, and selected parameters were not available from each patient. Therefore, measurement values differ from the n = 36 included patients. Blood glucose was assessed in the nonfasting state. Statistics: Depending on a non-Gaussian or Gaussian distribution, data are presented as median (with the range or as mean with standard deviation (SD)). Abbreviations: AAA, abdominal aortic aneurysm; ACE, angiotensin-converting enzyme; ARB, angiotensin II receptor blocker; ASA, acetylsalicylic acid; BMI, body mass index; CAD, coronary artery disease; CCB, calcium channel blockers; CRP, C-reactive protein; HDL, high-density lipoprotein; ILT, intraluminal thrombus; LDL, low-density lipoprotein; PAD, peripheral artery disease; SD, standard deviation; T2DM; type 2 diabetes mellitus; TC, total cholesterol; TG, triglycerides.

**Included patients, n**	**36**
**Clinical characteristics**	
Age—y, mean±SD, n	65.3±7.4, 36
Sex—m:f, % male	32:4, 89
Aortic diameter—mm, mean ± SD, n	62.8±13.9, 36
Thickness ILT—mm, median with range, n	17.5 (0.0–72.0), 32
CRP—mg/L, median with range, n	3.70 (0.50–127.3), 36
HDL—mmol/L, median with range, n	1.13 (0.65–2.43), 28
LDL—mmol/L, median with range, n	1.13 (0.71–6.79), 28
TC—mmol/L, mean ± SD, n	4.61±1.39, 28
TG—mmol/L, median with range, n	1.74 (0.77–4.28), 29
Blood glucose—mmol/L, median with range, n	5.38 (3.95–10.68), 31
**Pharmacological therapy**	
**ACE inhibitor—y:n, %**	**17:19, 47**
Ramipril, n/n total, %	13/17, 76
Lisinopril, n/n total, %	2/17, 12

Enalapril, n/n total, %	2/17, 12
**Anticoagulation—y:n, %**	**8:28, 22**
Rivaroxaban, n/n total, %	3/8, 38
Apixaban, n/n total, %	1/8, 13
Enoxaparin, n/n total, %	2/8, 25
Falithrom, n/n total, %	1/8, 13
**ARB—y:n, %**	**12:24, 33**
Valsartan, n/n total, %	7/12, 58
Candesartan, n/n total, %	5/12, 42
**ASA—y:n, %**	**20:16, 56**
**Beta-blocker—y:n, %**	**18:18, 50**
Metoprolol, n/n total, %	9/18, 50
Bisoprolol, n/n total, %	4/18, 22
Carvediol, n/n total, %	1/18, 6
Propranolol, n/n total, %	1/18, 6
Nebivolol, n/n total, %	2/18, 11
**CCB—y:n, %**	**15:21, 42**
Amlodipine, n/n total, %	12/15, 80
Nifedipine, n/n total, %	1/15, 7
Lercanidipine, n/n total, %	2/15, 13
**Diuretics—y:n, %**	**13:23, 36**
Toresamide, n/n total, %	4/13, 31
Furosemide, n/n total, %	1/13, 8
Hydrochlorothoazide, n/n total, %	7/10, 54
Indapamide, n/n total, %	1/10, 8
**Statin—y:n, %**	**26:10, 72**
Simvastatin, n/n total, %	5/26, 19
Atorvastatin, n/n total, %	18/26, 69
Rosuvastatin, n/n total, %	2/26, 8
**Cardiovascular risk factors**	
**BMI—kg/m², median with range, n**	27.0 (19.8–42.9), 36
**Smoking—y:n, %**	23:13, 64
**T2DM—y:n, %**	5:31, 14
**Hypertension—y:n, %**	31:5, 86
**CAD—y:n, %**	13:23, 36
**PAD, carotid artery stenosis—y:n, %**	11:25, 36

Table S3. Aortic HO-1 mRNA expression in response to cardiovascular risk factors in patients undergoing open surgical repair due to AAA. Data were analyzed by multiple linear regression, and HO-1 was set as the outcome variable. Data were log transformed, and one statistically significant outlier was omitted from the analysis. The estimate shows the proportional increase in HO-1 expression if the patient had the corresponding risk factor (= 1), whereas those that did not have the corresponding risk factor serve as the reference level (= 0). The proportional increase in HO-1 expression with age and total cholesterol concentrations is given per unit (years, mmol/L). Abbreviations: CAD, coronary artery disease, CI, confidence interval.

Risk factor	Estimate	CI left	CI right	p-value
Smoking [0]	0.722	0.144	3.625	0.700
Hypertension [0]	0.937	0.239	3.678	0.928
Age, years	0.988	0.898	1.086	0.802
CAD [0]	0.493	0.148	1.642	0.273
Total cholesterol, mmol/L	0.872	0.589	1.290	0.506

Table S4. Aortic HO-1 protein expression in response to cardiovascular risk factors in patients undergoing open surgical repair due to AAA. Data were analyzed by multiple linear regression, and HO-1 was set as the outcome variable. Data were log transformed, and one statistically significant outlier was omitted from the analysis. The estimate shows the proportional increase in HO-1 expression if the patient had the corresponding risk factor (= 1), whereas those that did not have the corresponding risk factor serve as the reference level (= 0). The proportional increase in HO-1 expression with age and total cholesterol concentrations is given per unit (years, mmol/L). Abbreviations: CAD, coronary artery disease, CI, confidence interval.

Risk factor	Estimate	CI left	CI right	p-value
Smoking [0]	1.956	0.333	11.471	0.467
Hypertension [0]	0.600	0.127	2.827	0.526
Age, years	1.076	0.966	1.200	0.199
CAD [0]	2.268	0.593	8.684	0.247
Total cholesterol, mmol/L	0.908	0.560	1.473	0.700
